# Evaluation of the efficacy of clear aligners in mandibular advancement and their effect on mandibular trabecular structures using fractal dimension analysis

**DOI:** 10.1186/s12903-025-05925-3

**Published:** 2025-08-21

**Authors:** Nurver Karslı, Merve Gonca, Gizem Mine Türksever, Nesrin Atasaral Beyaz

**Affiliations:** 1 Faculty of Dentistry, Department of Orthodontics, Karadeniz Technical University, Trabzon, Turkey; 2 Faculty of Dentistry, Department of Orthodontics, Eskişehir Osmangazi University, Eskişehir, Turkey; 3 Private Dental Clinic, Trabzon, Turkey

**Keywords:** Class II malocclusion, Clear aligners, Mandibular advancement, Fractal dimension analysis, Panoramic radiography

## Abstract

**Background:**

The objective of this study was to evaluate the efficacy of the mandibular advancement protocol (MA) with clear aligners in the treatment of skeletal Class II malocclusion and to assess their effect on the mandibular trabecular structure using fractal dimension analysis (FD-A) on panoramic radiographs.

**Methods:**

The study included panoramic and cephalometric radiographs of 20 patients (treatment group, mean age: 11.7 ± 0.8 years; 9 girls, 11 boys) with Class II malocclusion, treated with the mandibular advancement protocol (MA) using clear aligners. The radiographs were obtained before treatment (T0) and following the MA phase (T1). Additionally, panoramic radiographs of 20 control subjects who had undergone no orthodontic treatment (control group, mean age: 11.7 ± 0.9 years; 9 girls, 11 boys) were analyzed. The fractal dimension values of the mandibular trabecular structures in the condylar, gonial, and corpus regions were evaluated for both groups. Statistical analyses were performed using paired t-test and independent samples t-test for parametric data, and Wilcoxon test and Mann-Whitney U test for non-parametric data. Statistical significance was set at *P* < 0.05.

**Results:**

The treatment applied using MA with aligners resulted in significant decreases in SNA (*P* < 0.05), ANB (*P* < 0.05), 1-NA (*P* < 0.001), overjet (*P* < 0.001), and overbite (*P* < 0.01). Conversely, significant increases were observed in S-Go (*P* < 0.01) and 1-NB (*P* < 0.05) parameters. The FD-A changes between T0 and T1 indicated that the observed increases in the right and left condylar processes, the left gonial region, and the left mandibular corpus were not statistically significant in the treatment group (*P* > 0.05). The comparison of the FD-A results between the groups revealed no statistically significant differences (*P* > 0.05).

**Conclusions:**

The FD-A results revealed no significant alterations in the trabeculation of the mandibular bone in the treatment group. These findings suggest that MA treatment with aligners primarily contributes to dental correction rather than inducing changes in the skeletal structure and trabeculation of the mandibular bone.

## Introduction

Skeletal Class II malocclusion is among the most prevalent and clinically significant skeletal discrepancies encountered in orthodontics, affecting a considerable proportion of individuals with dentofacial deformities. This condition is primarily characterized by the retrognathic positioning of the mandible in relation to the maxilla, resulting in an abnormal sagittal relationship between the upper and lower jaws [1].

The widespread occurrence of skeletal Class II malocclusion has stimulated extensive research into its underlying etiological factors and the development of effective treatment strategies. A key objective in managing this condition is to achieve a balanced relationship between the maxilla and mandible, thereby improving both functional and aesthetic outcomes. In this regard, many clinicians have concentrated on harnessing the growth potential of the mandible and condylar region to promote forward mandibular growth and restore an optimal sagittal relationship between the jaws [2].

A range of treatment modalities has been developed for Class II malocclusions according to the specific subtypes of malocclusion. These include fixed and removable functional appliances, extraoral traction, maxillary expansion, fixed multibracket therapy, and intermaxillary elastics [3–5].

Mandibular retrognathia has a significant impact on quality of life, particularly in terms of aesthetic appearance [6]. The severity of the skeletal malocclusion is a key factor in determining the most appropriate treatment strategy. Orthodontic treatment with functional appliances such as the Activator, Twin-block (TB) and Herbst are widely used to correct jaw growth towards a more optimal relationship. In addition to selecting the appropriate treatment strategy, intervening at the right time in patients with mandibular retrognathia is another crucial factor for achieving optimal outcomes. Functional appliances are particularly effective in individuals with continued growth potential, such as adolescents, with the goal of achieving favourable aesthetic outcomes [7–12]. Several radiographic techniques are employed to accurately determine the optimal timing for treatment.

The cervical vertebral maturation (CVM) method has been demonstrated to be a highly reliable diagnostic tool for assessing growth and development. Its application in orthodontic decision-making has been shown to enhance treatment outcomes by combining effective and efficient protocols with optimal treatment timing. Numerous studies have compared the validity of the CVM method to hand-wrist radiography, with results confirming the high reliability of the CVM method. Furthermore, several studies have highlighted the advantages of the CVM method, including the elimination of the need for additional radiographs and a reduction in the total radiation dose exposure to patients [13–16].

However, the unwieldy design of functional appliances, in conjunction with the manifold challenges inherent in their fabrication — including multiple laboratory stages, discomfort during use, and technological advancements — have prompted orthodontists to seek alternative solutions [12].

Recent years have seen a significant increase in the development of various orthodontic appliance designs intended to meet the aesthetic expectations of pre-adolescent and adolescent patients. The F22® Young device, for instance, has been specifically designed to enhance aesthetic appeal, thereby improving patient compliance, while simultaneously providing orthopedic effects to stimulate mandibular advancement [17]. Moreover, clear aligner therapy has emerged as a highly preferred treatment modality among contemporary orthodontic approaches, offering advantages such as improved oral hygiene, enhanced periodontal health, and a reduced risk of root resorption, in addition to meeting aesthetic objectives [18, 19]. In addition, Align Technology™ (San José, CA, USA) has introduced the Invisalign Mandibular Advancement technique, which facilitates anterior mandibular advancement through the use of precision wings integrated into the aligners. These precision wings, located along the buccal aspect of the aligners, have been demonstrated to encourage anterior mandibular growth while concurrently aligning the teeth [20–23]. The efficacy of this appliance in addressing various dental concerns, including rotational misalignments prior to mandibular advancement, as well as the leveling and, when necessary, proclination of the incisors, has been well-documented [21].

Fractal dimension analysis (FD-A) is a mathematical technique used to measure the complexity of structures based on fractal dimensions [24–26]. This method is particularly valuable for evaluating changes in trabecular bone architecture, whether due to disease progression or treatment effects, offering the advantages of being both accessible and non-invasive [25, 27, 28]. In recent years, the application of FD-A has significantly expanded, with growing support for its use as an objective diagnostic tool in dentistry, particularly for assessing trabecular bone structure through commonly used dental panoramic radiographs (PRs) [25, 26]. Furthermore, recent studies have evaluated the effects of bruxism, various systemic conditions and bisphosphonate use on mandibular trabecular structure through FD-A [29–31].

Given that pre- and post-treatment panoramic radiographs are routinely captured in orthodontic practice, FD-A can be conveniently applied to these images to assess the impact of orthodontic treatments on the bone architecture of the jaws. During orthodontic tooth movement, whether influenced directly or indirectly by functional forces, remodeling of the trabecular bone structure occurs [32]. Previous studies have highlighted that microstructural changes in trabeculation can significantly affect bone morphology [33]. A review of the current literature reveals several studies that have utilized FD-A to investigate alterations in trabecular bone structure in the jaws during orthodontic treatment [25, 26, 28, 34].

Although the skeletal and dentoalveolar effects of mandibular advancement (MA) with clear aligners have been evaluated, to our knowledge, no studies have investigated the trabecular structural effects of MA with clear aligners on the jaws during the pubertal growth phase using FD-A. Identifying potential changes in trabecular structure following MA with clear aligners in Class II malocclusion patients could provide valuable insights into the impact of the treatment method on bone architecture during pubertal development. Therefore, this study aims to investigate the trabecular effects of MA with clear aligners on mandibular structures using FD-A, as well as the skeletal and dentoalveolar effects through cephalometric analysis, in Class II malocclusion patients during pubertal growth phases.

The null hypotheses were the following:

1. There would be no difference in skeletal and dentoalveolar changes before and after the mandibular advancement phase with clear aligners.
2. There would be no difference in the fractal dimension values of the mandibular trabecular structure before and after the mandibular advancement phase with clear aligners.
3. There would be no difference in the fractal dimension values of the mandibular trabecular structure in time-dependent changes between the groups.

## Materials and methods

### Study design and ethical approval

The present retrospective single-center study was performed through the analysis of lateral cephalometric radiographs and panoramic radiographs from clinical archival records. Ethics committee approval was obtained from the Karadeniz Technical University Faculty of Dentistry Scientific Research Ethics Committee (Approval Date: 21 September 2022; Approval Number: 2023/10). Written informed consent forms, routinely obtained prior to the commencement of treatment and encompassing consent for the use of patient records in scientific research, were obtained from the parents or legal guardians of participants under the age of 16.

### Sample size calculation and groups

The power of the sample size was calculated using the G*Power 3.1 software (Heinrich-Heine University of Dusseldorf, Germany). The effect size for the sample size calculation in this study was derived from the mean and standard deviation values of the buccoapical FD-A measurements in the monoblock group between T0 and T1. Based on the calculated effect size of 1.1744404 and a 5% α error probability, the statistical power of the study was determined to be 95%. Accordingly, the required total sample size was calculated as 40 patients, with 20 patients allocated to each group (critical t-value: 2.0243942; non-centrality parameter: 3.7139066) [34]. Therefore, the control group (mean age: 11.7 ± 0.9 years; 9 girls, 11 boys) included 20 individuals who were subjected to routine dental procedures at two different time points, with two PRs collected, to analyze the normal changes occurring in mandibular trabecular structures due to growth, in comparison to the treatment group. Pre- (T0) and post-mandibular advancement phase (T1) digital lateral cephalometric radiographs (LCRs) and PRs of 20 patients (treatment group, mean age: 11.7 ± 0.8 years; 9 girls, 11 boys), whose MA phase treatment with aligners was completed between 2020 and 2022 and who met the following inclusion criteria, were also included in the study.

### Inclusion and exclusion criteria

The inclusion criteria for the treatment group were defined as follows:

- High-quality pre- and post-mandibular advancement phase radiographic images.
- Pre-treatment skeletal Class II malocclusion due to mandibular deficiency (ANB ≥ 4° and SNB ≤ 80°).
- Being at peak pubertal growth period (CVM stage, CS3) [15, 16].
- Being treated with the MA protocol using clear aligners.
- Good cooperation with aligner wear throughout the MA phase.

All ClinCheck treatment plans were formulated by an experienced orthodontist and applied to the patients.

The control group was selected based on the following inclusion criteria: similar age, gender, and comparable time intervals between T0 and T1 for the obtained panoramic radiographs (PRs) to those of the treatment group; presentation at the clinic for routine dental treatment; and the absence of a history of orthodontic treatment, systemic diseases, or craniofacial syndromes. The absence of lateral cephalometric radiographs (LCRs) in the control group precluded the performance of a cephalometric analysis.

Patients who did not meet any of the inclusion criteria in all groups were excluded from the study.

### Mandibular advancement protocol with clear aligners

The technique of MA with clear aligners consists of a series of aligners designed to fit each dental arch. The buccal projections on the maxillary and mandibular aligners, referred to as precision wings, are made from the same material as the aligners and serve to gradually advance the mandible forward [20, 21]. The aligners are responsible for tooth movements, while the two precision wings maintain the mandible in a forward position [20]. It is designed to function similarly to other tooth-borne removable functional appliances for growth modification in patients with skeletal Class II malocclusion [35].

The patients in the treatment group were treated until the molars achieved a Class I relationship or the overjet was corrected to an edge-to-edge position in centric relation. During the advancement phase, the mandible was advanced sequentially by 2 mm every 8 weeks until the incisors were edge-to-edge [36]. The MA protocol involved changing the aligner weekly and included its use for at least 20–22 h per day.

### Cephalometric measurements

**Fig. 1:**
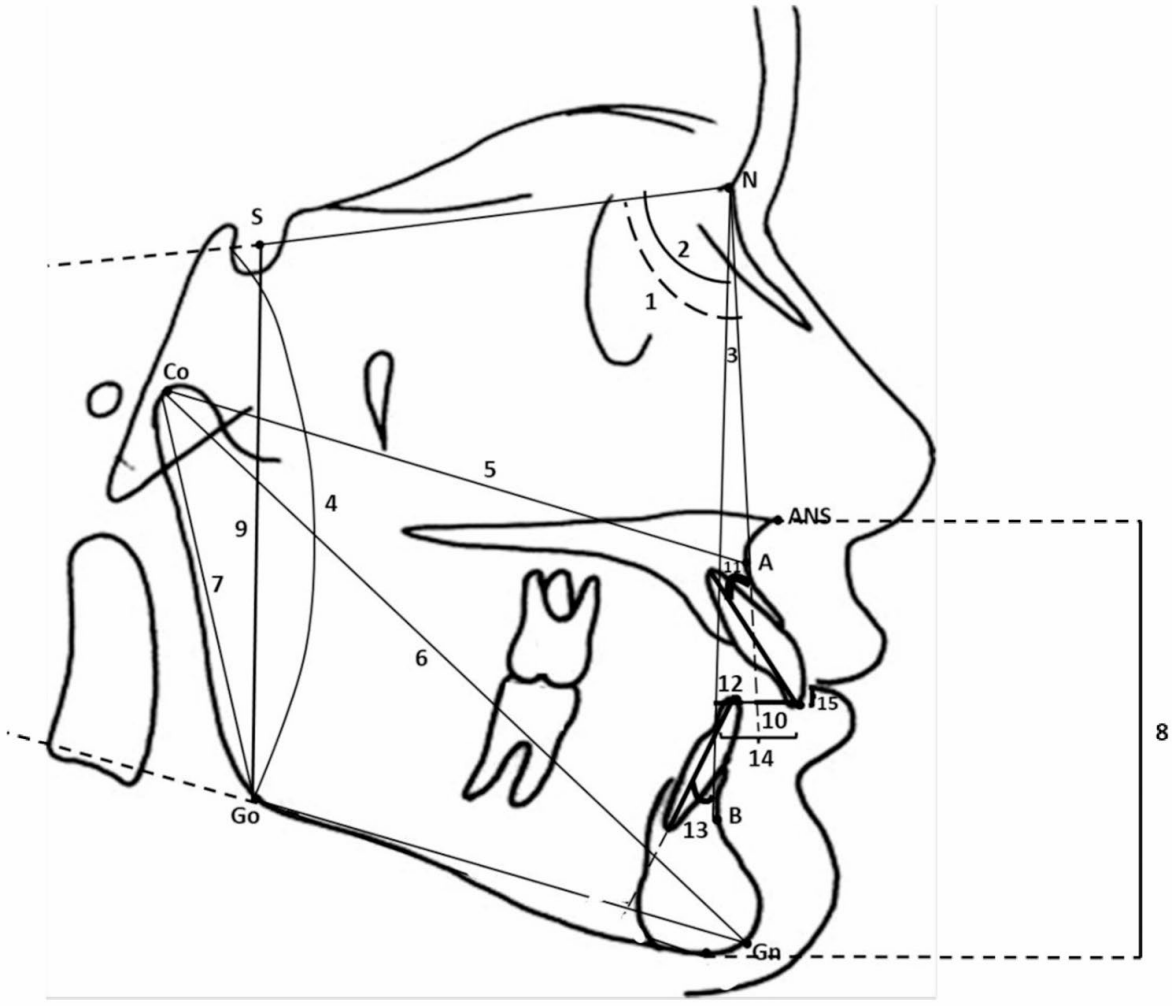
Cephalometric measurements. Skeletal angular measurements (°): (1) SNA; (2) SNB; (3) ANB; (4) SN-GoGn. Skeletal linear measurements (mm): (5) Co-A; (6) Co-Gn; (7) Co-Go; (8) ANS-Me; and (9) S-Go. Dentoalveolar measurements: (10) 1-NA (mm); (11) 1-NA (°); (12) 1-NB (mm); (13) 1-NB (°); (14) overjet (mm); (15) overbite (mm)

LCRs for the treatment group were obtained using the Kodak 9000 Extraoral Imaging System (Carestream Health, Inc., Rochester, NY, USA) with imaging parameters set to 64 kVp, 15 mA, and an exposure time of 0.4 s. Cephalometric measurements were performed using NemoCeph software (NemoStudio 2022, Software Nemotec S.L.) and included four skeletal angular measurements, six skeletal linear measurements and six dentoalveolar measurements, as shown in Table 1; Fig. 1.

**Table 1:** Definitions of cephalometric measurements utilized

Measurements	Definition
Skeletal angular measurements (°)	
SNA (°)	Angle between sella, nasion and point A
SNB (°)	Angle between sella, nasion and point B
ANB (°)	Angle between point A, nasion and point B
SN-GoGn (°)	Angle between SN and gonion-gnathion line
Skeletal linear measurements (mm)	
Co-A (mm)	Distance between condylion and point A
Co-Gn (mm)	Distance between condylion and gnathion
Co-Go (mm)	Distance between condylion and gonion
ANS-Me (mm)	Distance between anterior nasal spine and menton
S-Go (mm)	Distance between sella and gonion
Dental measurements	
1-NA (°)	Angle of the long axis of the maxillary incisor to nasion-point A line
1-NA (mm)	Distance between the tip of the maxillary incisor and nasion-point A line
1-NB (°)	Angle of the long axis of the mandibular incisor to nasion-point B line
1-NB (mm)	Distance between the tip of the mandibular incisor and nasion-point B line
Overjet (mm)	Horizontal distance between the incisal surface of the upper central incisor and the labial surface of the lower central incisor
Overbite (mm)	Vertical distance by which the incisal edge of the upper central incisor overlap the labial surface of the lower central incisor

### Fractal dimension analysis on panoramic radiographs

All PRs were taken using the Sirona Orthophos XG 5 device with a resolution of 0.027 mm pixel size and imaging parameters set to 64 kVp, 8 mA and an exposure time of 8.0 s. According to the manufacturer’s dose specifications, the dose area product (DAP) was recorded as 39 mGy-cm².

The FD-A was performed using ImageJ software (version 1.52a, US National Institutes of Health) according to the method described by White and Rudolph [37]. The box-counting algorithm was used to analyse selected regions of interest (ROIs) in the mandible. ROIs of 50 × 50 pixels were identified at three different locations on the right and left sides of the mandible by an experienced orthodontist.

Region 1: Condylar process, referring to the condylar area located below the cortex.

Region 2: Angulus mandibula, representing the supra-cortical region in the gonial area.

**Fig. 2:**
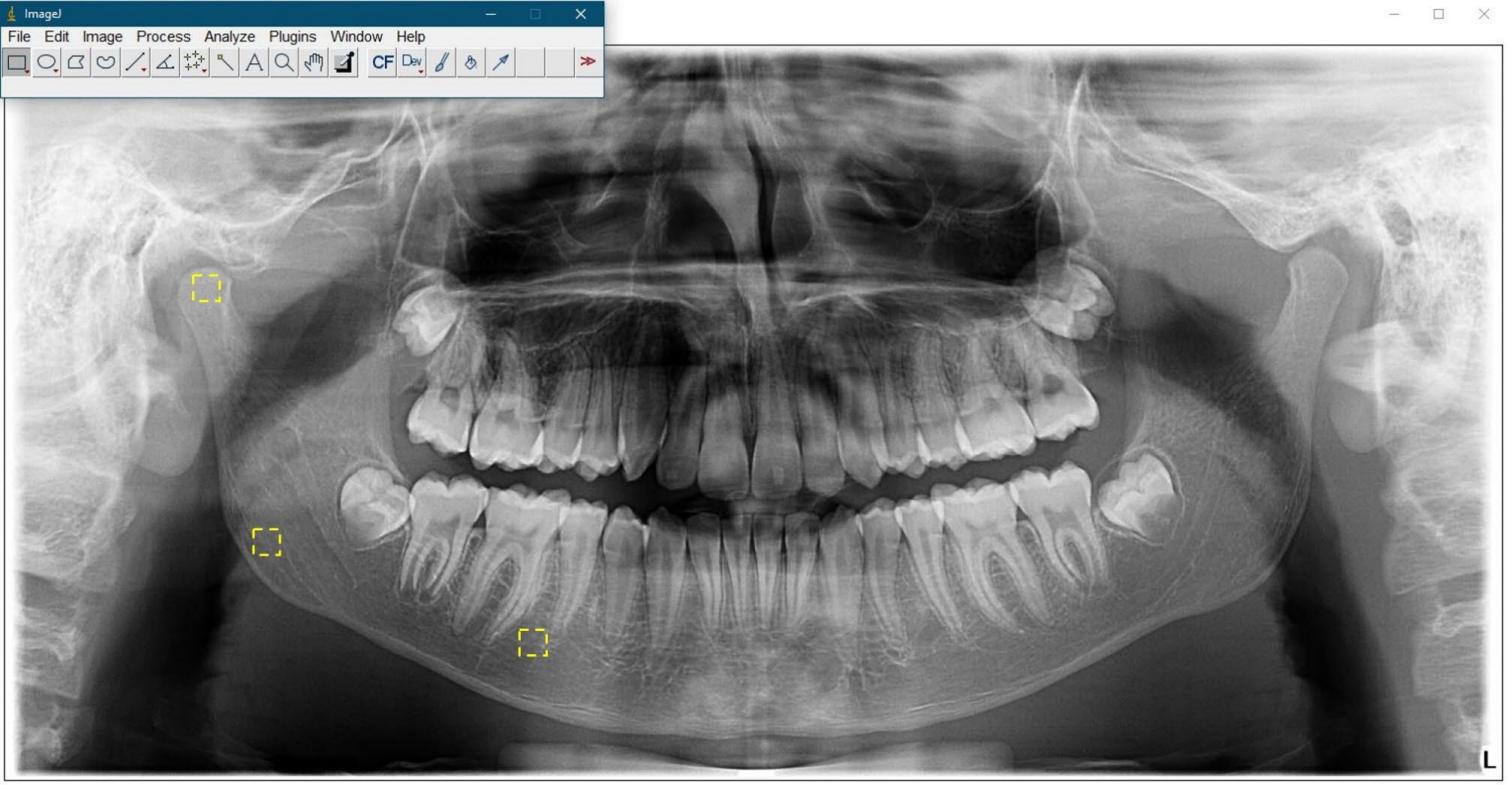
Regions of interest (ROIs) from three different areas in the mandible (condylar process, angulus mandibula, corpus mandibula)

Region 3: Corpus mandibula, encompassing the area between the second premolar and the first molar, situated above the mandibular canal (Fig. 2).

**Fig. 3:**
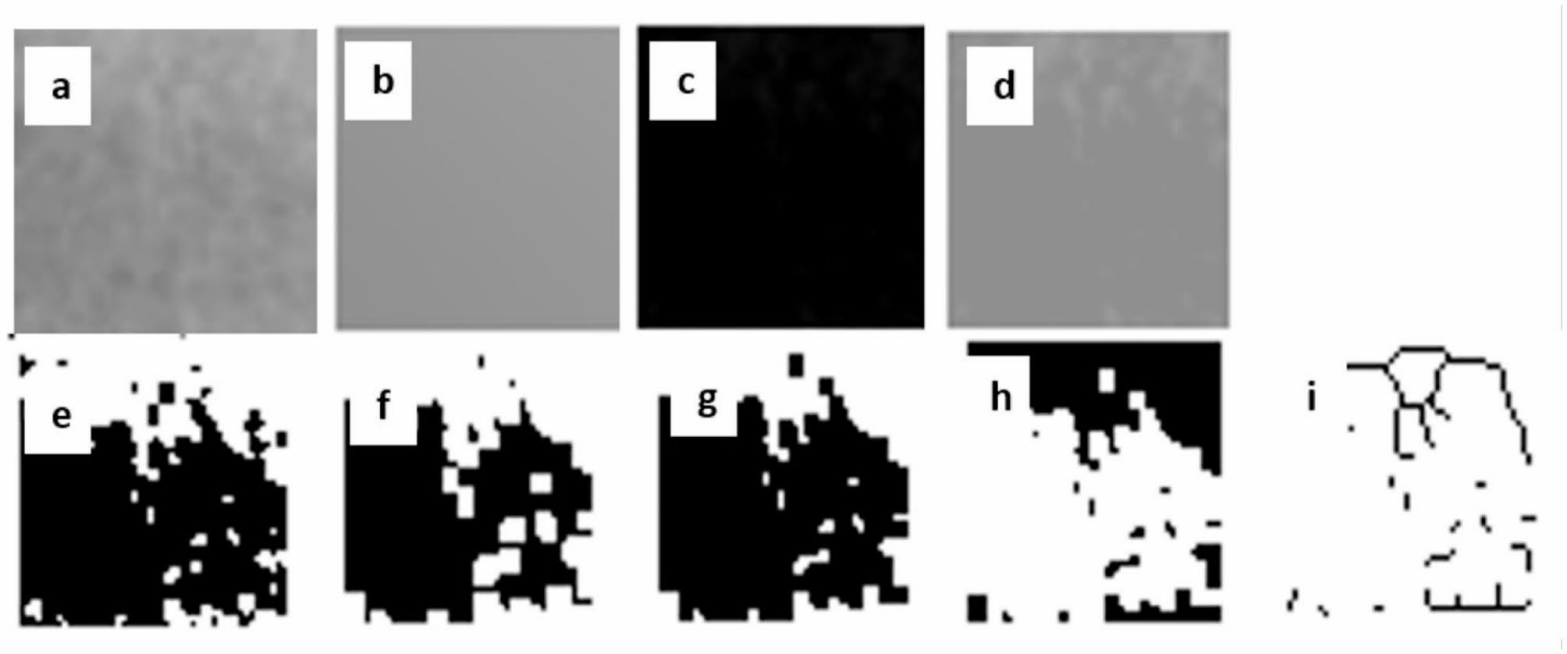
Steps of fractal dimension analysis (FD-A): **A**: cropped image, **B**: addition of the Gaussian blur filter, **C**: subtraction of the blurred image from the cropped image, **D**: addition of a gray value of 128 to each pixel to separate bone marrow spaces from trabeculae, **E**: binary conversion to a black and white format with thresholding and a brightness value of 128, **F**: eroded image, **G**: dilated image, **H**: inverted image, **I**: skeletonized image

The high-resolution PRs of patients in both groups were converted into tagged image file formats (TIFFs). The regions of interest (ROIs) were identified and duplicated. To minimize brightness variations, the duplicated images were processed using a Gaussian blur filter, and the blurred versions were subtracted from the original images. To differentiate between bone marrow spaces and trabeculae, each pixel was assigned a uniform gray value of 128. Thresholding was applied to produce binary (black-and-white) images, followed by noise reduction using the erosion function. Subsequent steps included dilation, inversion, and skeletonization, which preserved only the central parts of the trabeculae. The images were then segmented into frames of varying sizes (2, 3, 4, 6, 8, 12, 16, 32, and 64 pixels), and the total number of frames in each image was calculated. As a final point, FD-A values, which quantify structural complexity, were determined (Fig. 3).

### Measurement error

To assess the reliability of LCR measurements and FD-A values, the same orthodontists repeated the measurements on twenty randomly selected individuals 4 weeks after the initial evaluation using the intraclass correlation coefficients (ICC) [38, 39].

### Statistical analysis

Data were analysed using IBM SPSS Statistics 21.0 software (SPSS, Chicago, IL, USA). Descriptive statistics for quantitative variables were calculated and presented in tables, including mean, standard deviation, median, minimum and maximum values. Normality of data distribution was assessed using the Shapiro-Wilk test. Paired samples t-tests were used to assess changes before and after treatment for dependent variables with parametric distribution, and the Wilcoxon signed rank test for non-parametric variables. Comparisons between groups were performed using the independent samples t-test for parametric data and the Mann-Whitney U test for non-parametric data. A *p*-value < 0.05 was considered statistically significant.

## Results

LCR and FD-A measurements were repeated by the same orthodontist 4 weeks after the initial assessments to evaluate intraobserver reliability. The intraclass correlation coefficients (ICC) demonstrated excellent reliability, ranging from 0.900 to 0.998 for LCR measurements and from 0.976 to 0.996 for FD-A values [38, 39].

The total sample consisted of 40 patients divided into two groups: the treatment group (20 patients: 9 girls, 11 boys; mean age: 11.7 ± 0.8 years) and the control group (20 patients: 9 girls, 11 boys; mean age: 11.7 ± 0.9 years). No statistically significant differences in age distribution were observed between the two groups at T0 (*P* = 0.912). Cephalometric data for all patients in the treatment group at the beginning of treatment indicated that they were in the CS3 cervical vertebral maturation stage.

The mean duration of treatment (T1-T0) was 1.12 ± 0.2 years for the treatment group and 1.1 ± 0.2 years for the control group. There were no statistically significant differences in treatment duration between the groups (*P* = 0.858).

### Cephalometric measurements

Analysis of the changes in cephalometric measurements from T0 to T1 in the treatment group revealed a significant decrease in both the SNA and ANB angles (*P* < 0.05) and a significant increase in the S-Go distance (*P* < 0.01). For dentoalveolar measurements, a significant decrease in the 1-NA angle (*P* < 0.001) and a significant increase in the 1-NB angle (*P* < 0.05) were observed. In addition, significant decreases in both overjet (*P* < 0.001) and overbite (*P* < 0.01) were observed after treatment (Table 2).

**Table 2:** Descriptive statistics of cephalometric parameters and comparison of the cephalometric changes occurred during Post-MA phase (T1) and Pre-treatment (T0) for treatment group

	Pre-treatment (T0)	Post-treatment (T1)		T1-T0				
Parameters	Mean ± SD	Mean ± SD		Mean ± SD			*P*-Value	
Skeletal angular measurements (°)								
SNA	83.00 ± 3.77	82.69 ± 3.42		-0.31 ± 0.61			**0.044** ^§^	
SNB	77.38 ± 3.28	77.44 ± 3.17		0.06 ± 0.44			0.539^§^	
ANB	5.62 ± 1.60	5.25 ± 1.65		-0.37 ± 0.62			**0.016** ^§^	
SN-GoGn	28.75 ± 4.26	29.19 ± 4.2		0.44 ± 1.13			0.101^§^	
Skeletal linear measurements, (mm)								
Co-A	80.05 ± 3.99	79.94 ± 5.01		-0.11 ± 2.15			0.819^§^	
Co-Gn	98.95 ± 6.35	99.07 ± 5.76		0.13 ± 1.41			0.692^¶^	
Co-Go	50.95 ± 4.23	51.57 ± 4.3		0.62 ± 1.58			0.096^§^	
ANS-Me	57.16 ± 6.01	57.36 ± 6.07		0.2 ± 0.88			0.325^§^	
S-Go	68.66 ± 5.73	69.76 ± 6.13		1.1 ± 1.47			**0.003** ^§^	
Dentoalveolar measurements								
1-NA (mm)	3.57 ± 3.53	3.42 ± 1.98		-0.15 ± 2.04			0.749^§^	
1-NA (°)	29.23 ± 5.55	25.6 ± 5.51		-3.63 ± 1.31			**< 0.000** ^§^	
1-NB (mm)	2.78 ± 1.96	3.26 ± 1.08		0.47 ± 1.67			0.220^§^	
1-NB (°)	23.06 ± 5.38	23.68 ± 5.11		0.62 ± 0.98			**0.011** ^§^	
Overjet	6.77 ± 1.89	4.35 ± 1.42		-2.42 ± 1.15			**< 0.000** ^§^	
Overbite	4.27 ± 1.73	3.17 ± 1.09		-1.1 ± 1.35			**0.006** ^¶^	

MA; Mandibular Advancement Technique with Aligners, Min.; Minimum, Med.; Median, Max.; Maximum, SD; Standard deviation

^§^; Paired sample t test, ^¶^; Wilcoxon signed rank test, *P* < 0.05 considered statistically significant

### Fractal dimension analysis

The treatment group showed higher values in the left mandibular corpus region compared to the control group at T0, with a statistically significant difference (*P* < 0.01; Table 3).

**Table 3:** Comparison of the mean values of the fractal dimension analysis (FD-A) parameters at the beginning of the observation period (T0) between the groups

	MA (T0)	Control (T0)	MA-Control (Diff.) (T0)	
	Mean ± SD	Mean ± SD	Mean ± SD	*P*-Value
Age (T0)	11.7 ± 0.8	11.7 ± 0.9	-0.03 ± 0.1	0.912
CO_L	1.414 ± 0.098	1.357 ± 0.131	0.056 ± 0.045	0.132^**†**^
CO_R	1.402 ± 0.107	1.391 ± 0.107	0.011 ± 0.037	0.745^**†**^
GO_L	1.387 ± 0.075	1.342 ± 0.108	0.045 ± 0.036	0.131^**†**^
GO_R	1.385 ± 0.063	1.344 ± 0.114	0.041 ± 0.033	0.330^‡^
CM_L	1.437 ± 0.058	1.366 ± 0.088	0.071 ± 0.019	**0.005** ^**†**^
CM_R_	1.408 ± 0.083	1.386 ± 0.086	0.021 ± 0.03	0.387^‡^

MA; Mandibular Advancement Technique with Aligners, CO; Condylar process, GO; Angulus mandibula, CM; Corpus mandibula, L; Left, R; Right, Min.; Minimum, Med.; Median, Max.; Maximum, SD; Standard deviation

^**†**^; Independent sample t test, ^‡^; Mann Whitney U test, *P* < 0.05 considered statistically significant

The alterations in FD-A values from T0 to T1 for the MA and control groups are illustrated in Tables 4 and 5, respectively. No statistically significant differences were observed in the FD-A values for either group (*P* > 0.05; Tables 4 and 5).

**Table 4:** Fractal dimension analysis (FD-A) changes and comparison of the changes occurring during post-MA phase (T1) and Pre-treatment (T0) for treatment group

	MA (T0)	MA (T1)	MA T1-T0 (Diff.)	
	Mean ± SD	Mean ± SD	Mean ± SD	*P*-Value
Age, y ^α^	11.7 ± 0.8	12.8 ± 0.9	1.12 ± 0.2	**0.000**
CO_L	1.414 ± 0.098	1.422 ± 0.078	0.008 ± 0.069	0.602^§^
CO_R	1.402 ± 0.107	1.425 ± 0.069	0.023 ± 0.097	0.297^§^
GO_L	1.387 ± 0.075	1.393 ± 0.074	0.006 ± 0.110	0.794^§^
GO_R	1.385 ± 0.063	1.368 ± 0.107	-0.017 ± 0.121	0.525^§^
CM_L	1.437 ± 0.058	1.440 ± 0.062	0.002 ± 0.070	0.876^§^
CM_R	1.408 ± 0.083	1.406 ± 0.076	-0.002 ± 0.106	0 0.478^¶^

MA; Mandibular Advancement Technique with Aligners, CO; Condylar process, GO; Angulus mandibula, CM; Corpus mandibula, L; Left, R; Right, Min.; Minimum, Med.; Median, Max.; Maximum, SD; Standard deviation

^§^; Paired sample t test, ^¶^; Wilcoxon signed rank test, *P* < 0.05 considered statistically significant

**Table 5:** Fractal dimension analysis (FD-A) changes and comparison of the changes occurring during post- (T1) and Pre-observation (T0) periods for control group

	Control (T0)	Control (T1)		Control T1-T0 (Diff.)		
	Mean ± SD	Mean ± SD		Mean ± SD	*P*-Value	
Age ^α^	11.7 ± 0.9	12.8 ± 1		1.1 ± 0.2	**0.000**	
CO_L	1.357 ± 0.131	1.371 ± 0.144		0.014 ± 0.187	0.743^§^	
CO_R	1.391 ± 0.107	1.392 ± 0.127		0.001 ± 0.136	0.983^§^	
GO_L	1.342 ± 0.108	1.341 ± 0.121		0.000 ± 0.116	0.991^§^	
GO_R	1.344 ± 0.114	1.347 ± 0.138		0.003 ± 0.081	0.823^¶^	
CM_L	1.366 ± 0.088	1.368 ± 0.101		0.002 ± 0.045	0.737^§^	
CM_R	1.386 ± 0.086	1.386 ± 0.115		-0.001 ± 0.082	0.575^§^	

CO; Condylar process, GO; Angulus mandibula, CM; Corpus mandibula, L; Left, R; Right, Min.; Minimum, Med.; Median, Max.; Maximum, SD; Standard deviation

^§^; Paired sample t test, ^¶^; Wilcoxon signed rank test, *P* < 0.05 considered statistically significant

A comparison of the time-dependent changes between the treatment and control groups revealed no statistically significant difference in FD-A values (*P* > 0.05; Table 6).

**Table 6:** Comparison of the post-observation (T1) - pre-observation (T0) differences between the groups

	MA (T1-T0)		Control (T1-T0)	MA-Control (Diff.)			
	Mean ± SD		Mean ± SD	Mean ± SD		*P*-Value	
CO_L	0.008 ± 0.069		0.014 ± 0.187	-0.006 ± 0.045		0.899^**†**^	
CO_R	0.023 ± 0.097		0.001 ± 0.136	0.023 ± 0.037		0.547^**†**^	
GO_L	0.006 ± 0.110		0.000 ± 0.116	0.007 ± 0.036		0.850^**†**^	
GO_R	-0.017 ± 0.121		0.003 ± 0.081	-0.02 ± 0.033		0.541^**†**^	
CM_L	0.002 ± 0.070		0.002 ± 0.045	0.000 ± 0.019		0.980^**†**^	
CM_R	-0.002 ± 0.106		-0.001 ± 0.082	-0.001 ± 0.03		0.871^‡^	

MA; Mandibular Advancement Technique with Aligners, CO; Condylar process, GO; Angulus mandibula, CM; Corpus mandibula, L; Left, R; Right, Min.; Minimum, Med.; Median, Max.; Maximum, SD; Standard deviation

^**†**^; Independent sample t test, ^‡^; Mann Whitney U test, *P* < 0.05 considered statistically significant

## Discussion

The objective of this retrospective study was to provide data on the skeletal effectiveness of mandibular advancement with aligners on mandibular trabecular bone. A review of the literature reveals conflicting findings regarding the primary effects of this technique. Some studies suggest that this treatment option primarily produces skeletal effects, while others emphasize the occurrence of predominantly dental effects [22, 23, 40, 41]. Given the absence of consensus on this matter, the present study aims to examine mandibular trabecular alterations through the use of FD-A on PRs in growing patients presenting with Class II malocclusion due to mandibular retrognathia, as well as to investigate the skeletal and dentoalveolar effects of MA with aligners using LCRs [23, 25, 36, 40]. Furthermore, as this treatment approach represents a contemporary method, it is anticipated that this study will contribute valuable insights to the existing literature and provide clinicians with a deeper understanding of the effects of mandibular advancement with aligners, particularly in terms of both skeletal and dentoalveolar outcomes.

Micro-computed tomography (micro-CT) and cone-beam computed tomography (CBCT) are considered the gold standard in bone structure evaluation; however, due to their high radiation exposure, they are not routine methods used for clinical diagnosis [42, 43]. Therefore, in this study, PRs, which are part of routine diagnostic records obtained from orthodontic patients, were used to quantitatively assess mandibular bone structure through FD-A.

FD-A allows changes in bone density to be expressed as mathematical data through measurements made on dental radiographs, without the need for invasive procedures [44]. In this study, FD-A was used to evaluate trabecular mineralization changes in mandibular bone structures related to growth in patients treated with MA using clear aligners and individuals in the control group. In this regard, the present study is the first to investigate the effects of MA with clear aligners on mandibular bone structures using FD-A.

A systematic review by Yu et al. found that most studies demonstrated effective mandibular advancement during the adolescent growth spurt [22]. Additionally, Wu et al. and Sabouni et al. confirmed that similar results could be achieved following MA treatment with clear aligners [23, 40].

Similarly, the treatment group in our study comprised patients who initiated treatment during the third stage of cervical vertebral maturation (CS3), corresponding to the peak pubertal growth phase, which is considered the optimal timing for functional jaw orthopedics [15, 16].

The study primarily involved separate fractal analyses conducted for the treatment and control groups. The findings revealed no statistically significant difference in the changes in FD-A values of the mandibular trabecular structure between the two groups. When considered in conjunction with the cephalometric findings, the analyses mutually supported each other in the treatment group. The time-dependent cephalometric results indicated that dentoalveolar changes were more pronounced than skeletal changes.

The cephalometric analysis of the treatment group demonstrated a significant decrease in ANB angle, primarily resulting from a decrease in SNA. These findings are consistent with, though slightly smaller than, the reductions in SNA (1.01°) and ANB (1.50°) reported by Hosseini et al. [36]. The significant decrease in SNA observed in the treatment group may be linked to the influence of upper incisor torque on the position of point A.

In terms of mandibular growth assessment, no significant changes in mandibular dimensional parameters (Co-A, Co-Gn, Co-Go, Go-Me) or SNB angle were observed after treatment. These results are consistent with the SNB findings reported by Hosseini et al. and Ravera et al., but contrast with the increases in mandibular parameters reported in several other studies [23, 35, 36, 40, 45, 46].

In the treatment group, there was a decrease of 2.42 mm in overjet, with a significant proportion of this change attributable to dental alterations. This finding was more than the 2.3 mm decrease in overjet reported by Hosseini et al. with the mandibular advancement treatment [36]. The reduction in overjet was accompanied by a decrease in the 1-NA angle and an increase in the 1-NB angle. Morris et al. observed a statistically significant retraction of the upper incisors and a slight proclination of the lower incisors following treatment with TB appliance [47]. In contrast to TB, it was hypothesized that aligners would have a more beneficial effect on incisor inclination due to their design, which covers the tooth surfaces. Similarly, Hosseini et al. concluded that the MA appliance was more effective than the Herbst appliance in controlling mandibular incisor inclination [36]. Caruso et al. compared the dentoalveolar effects of the TB and MA with aligners, finding that the aligners effectively controlled incisor inclination [46]. However, contrary to the findings of previous studies, a significant increase in lower incisor inclination was observed during the mandibular advancement phase in this study. Moreover, consistent with our findings, several studies have reported a significant decrease in overbite [35, 36, 40, 46]. Therefore, when summarizing the cephalometric findings of this study, we can clinically conclude that the use of clear aligners with MA therapy primarily improves Class II malocclusion through dentoalveolar correction, rather than stimulating the skeletal development of the mandible in growing individuals. As a result, the first null hypothesis was rejected.

The present study observed a non-significant increase in the mandibular plane angle, consistent with the findings reported by Glaser et al. and Hosseini et al. [35, 36]. This can be attributed to the ability of aligners to control molar extrusion, likely due to the occlusal surfaces of the molars being covered by the aligner. These findings suggest that MA with aligners may be a more suitable option for the correction of Class II malocclusion cases with a dolichofacial skeletal pattern.

A substantial number of studies utilizing FD-A to evaluate the mandibular trabecular structure have reported significant alterations in FD-A values within the condylar and corpus regions of patients treated with functional appliances. These studies have also documented considerable changes associated with an increase in total mandibular length following treatment [28, 48]. In contrast, Gümüş et al. observed no changes in FD-A values within the condylar region after functional treatment, with alterations only noted in the corpus region [26]. In contrast, the present study did not identify any significant alterations in the mandibular trabecular structure following MA with aligners. The lack of notable alterations in these areas was an anticipated outcome when considered alongside the cephalometric measurements obtained in the study. At the same time, no differences were observed when comparing the time-dependent changes in fractal values between the groups. Therefore, the second and third null hypotheses of this study were confirmed.

A limitation of the present study is that FD-A was conducted using two-dimensional PRs. Although previous studies have demonstrated the high reliability of this method, future research utilising three-dimensional imaging could provide additional insights and further enhance the existing literature. However, this study provides orthodontists with valuable insights into the clinical prognosis associated with the changes in mandibular bone structures resulting from mandibular advancement using clear aligners.

## Conclusion

Cephalometric analysis revealed that, contrary to expectations, mandibular advancement (MA) treatment with clear aligners did not significantly impact mandibular development in pubertal individuals. The cephalometric findings indicated that notable dental changes were observed, particularly in the correction of Class II malocclusion, including maxillary incisor retroclination and a reduction in overjet due to mandibular incisor proclination.

FD-A revealed that mandibular advancement treatment with clear aligners did not induce significant changes in the mandibular trabecular structure during puberty. Similarly, the FD-A changes in trabecular organization over time between the groups were not statistically significant.

## Data Availability

The data that support the findings of this study are available from the corresponding author (NK) upon reasonable request.
